# Food Problem Notes

**Published:** 1918-03-02

**Authors:** 


### Food Problem Notes.

Captain Tallents has been working strenuously to explain difficulties and remove hindrances in the scheme of rationing in meat and butter which, beginning in London on February 25, will spread a month later over the whole country. From his remarks and those of other authorities at Grosvenor House, addressed to journalists and accredited speakers, the following are taken.

It is decided, after consultation with high medical authorities, to make concessions to two classes only of invalids. The tuberculous will be allowed up to 2½ lb. of meat and up to 1 lb. butter or margarine. Diabetic cases will be allowed up to 1½ lb. butter or margarine. A certificate from the doctor must be taken to the local Food Office.

The present supply of meat does not permit a raising of the meat ration for men doing heavy work—*e.g.*, working seven days a week at manual labour. But the case has been put to some bodies of those most severely tried, and they have agreed to accept the present conditions, the promise being given that rations for them will be increased so soon as supplies permit.

Only three of the four available coupons for the week may be spent at the shop of the registered butcher. If preferred, all coupons may be used to buy game, etc., elsewhere. But arrangements will be made by which butchers who chance to find themselves overstocked with meat likely to become damaged may sell on fourth coupons. Communal kitchens may probably benefit by any such surplus.

Concessions to schools holding cookery classes may be expected.

